# Negotiating Verification: International Diplomacy and the Evolution of Nuclear Safeguards, 1945–1972

**DOI:** 10.1080/09592296.2017.1420520

**Published:** 2018-01-17

**Authors:** Elisabeth Roehrlich

## Abstract

Nuclear safeguards have been an essential part of the global order since the beginnings of the nuclear age. The International Atomic Energy Agency [IAEA], an international bureaucracy that is supposed to be a non-political, technical institution administers this global nuclear safeguards regime. Even though safeguards have always been controversial, they have turned out to be the most enduring item in the international community’s toolbox to prevent or slow down the spread of nuclear weapons to non-nuclear states. This analysis shows that nuclear safeguards, whilst they survived the fall of the Iron Curtain, were a genuine invention of the Cold War. At the beginning of the nuclear age, there was an overall understanding that safeguards were not strong enough to prevent the global spread of nuclear weapons. It was only over the course of the late 1950s and 1960s that safeguards moved from the margins to the centre of diplomatic negotiations about global nuclear order. Newly declassified records from the IAEA Archives in Vienna offer insights into the evolution of early nuclear safeguards and suggest that negotiation patterns, proceedings, and settings affected the outcome of this nuclear diplomacy.

Nuclear safeguards have been an essential part of the global order since the beginnings of the nuclear age.^1^ They encompass different measures, such as inspections and material accountancy, to verify that states, which have signed international agreements not to use nuclear materials for weapons development, do in fact abide to these obligations.^2^ Probably the most widely known aspect of nuclear safeguards is the international inspectorate that carries out on-the-ground visits in member-states of the International Atomic Energy Agency [IAEA]. Some of today’s most heatedly discussed international challenges relate directly to the issue of nuclear safeguards. The international crises surrounding North Korea’s nuclear ambitions began in 1992, when that country provided the IAEA with a flawed initial report about its nuclear activities, thereby violating its safeguards agreement with the Agency.^3^ Iran’s commitments under the Joint Comprehensive Plan of Action [JCPOA], agreed upon in 2015 by the five permanent members of the United Nations [UN] Security Council and Germany, are verified and monitored by IAEA safeguards.^4^

Historically, and despite their widely accepted key role in international relations, nuclear safeguards have always been controversial. States feared—and continue to fear—that inspectors might conduct industrial espionage, and they often perceive the system as a threat to national sovereignty. Smaller states argue that international nuclear safeguards consolidate superpower hegemony because, under the Nuclear Non-Proliferation Treaty [NPT], nuclear weapon states do not have to accept safeguards on their own nuclear programmes. Yet, at the same time, nuclear safeguards are probably the most enduring item in the international community’s toolbox to prevent or slow down the spread of nuclear weapons to other states. As the most recent developments underline, international safeguards are still an essential, widely trusted, and somewhat flexible pillar of the non-proliferation regime.

This analysis shows that nuclear safeguards, whilst they survived the fall of the Iron Curtain, were a genuine invention of the Cold War.^5^ In November 1945, three months after the bombing of Hiroshima and Nagasaki, the United States, Canada, and Britain released the Three-Nation Agreed Declaration on the future control of nuclear energy. The development of the atomic bomb made these governments call for an international response to the challenges posed by the new weapon, which was incomparable in its destructiveness. To prevent states from developing national nuclear weapons capabilities—at that time the United States was the only atomic Power—they envisaged a system of “effective, reciprocal, and enforceable safeguards acceptable to all nations.”^6^ A decade later, in 1957, the international community launched the IAEA, a new international organisation linked to the UN system that, for the first time, established an international safeguards regime.^7^ The entry-into-force of the NPT in 1970 made nuclear safeguards mandatory for all non-nuclear weapon states that had signed the treaty.^8^ The IAEA, an international bureaucracy that is supposed to be a non-political and technical institution, administers these safeguards.^9^ From the 1950s through the early 1970s, diplomats and state officials negotiated the foundations of this international nuclear safeguards regime. Recently declassified IAEA records offer startling insights into these early diplomatic negotiations on international nuclear verification.^10^

What did the founders of the international safeguards regime expect them to accomplish? Did they believe that nuclear safeguards would have the power to prevent the proliferation of nuclear weapons? Alternatively, did the involved diplomats and officials envisage safeguards primarily as a confidence-building measure between and amongst states? In addition, how was it possible that amid Cold War tensions, the negotiators were successful in building the foundations of a lasting verification regime? Accordingly, three critical moments in nuclear safeguards diplomacy are crucial: the initial proposals on international control in 1945–1946, particularly American and Soviet proposals to the UN Atomic Energy Agency [UNAEC]; multi-national negotiations that produced the IAEA in 1953–1957; and, from the mid-1960s to the early 1970s, the negotiations that eventually made the IAEA the key verification institution of the NPT.

At the beginnings of the nuclear age, there was an over-all understanding that safeguards alone would not be able to prevent the global spread of nuclear weapons. Only over the course of the diplomatic negotiations in the late 1950s and 1960s did safeguards move from the margins to the centre of the global nuclear order; they became the core, rather than a supplement, of international efforts to reduce the threat posed by nuclear weapons. Moreover, the specific conditions of diplomacy—the settings and procedures of negotiations—had a decisive impact on their respective outcomes. In 1959, the American negotiator, Bernhard G. Bechhoefer, described the creation of the IAEA as a “unique episode in post–World War II diplomatic history.” Looking at the reasons for the success of IAEA negotiations, Bechhoefer concluded that in dealing with the Soviet Union, it was “necessary to place what would otherwise appear to be an undue emphasis on procedures.”^11^ Did other conditions—confidentiality level, size of delegations, professional expertise—shape the discussions; and to what extent did these leave an imprint on the nature of nuclear safeguards?

The IAEA has a dualistic architecture as it is both a diplomatic forum and technical bureaucracy.^12^ Its staff, the Secretariat, is composed of international civil servants headed by the Director General. Upon taking a position at the IAEA, each staff member has to declare that she or he will only act in the interest of the organisation and “not to seek or accept instructions” from any government.^13^ Next to the Secretariat, two diplomatic bodies control the fate of the Agency. Whilst the General Conference is the annual gathering of national delegations of all IAEA member-states, the smaller and more frequently convening Board of Governors is the Agency’s most powerful policy-making organ.^14^ This dualistic architecture of bureaucracy and diplomacy reflects the Agency’s safeguards activities. Whilst inspectors and analysts in the IAEA’s Department of Safeguards are usually international civil servants, diplomats and other member-state officials are in charge of deciding on the system’s regulations. Neither the IAEA statute, the NPT, nor any of the other safeguards regulations are self-enforcing; the IAEA can only conduct safeguards activities once it has negotiated and signed a safeguards agreement with the respective state.^15^

Whilst nuclear safeguards are primarily associated with the work of the IAEA—and have reached their most powerful force in this framework—the concept of international safeguards predates the creation of the Agency in 1957. The delegates of the 1919 Paris Peace Conference used the notion of safeguards when they anticipated a global order built on international co-operation and law. They proposed, “a League of Nations be created to promote international co-operation, to ensure the fulfilment of accepted international obligations, and to provide safeguards against war.”^16^ In 1945, the Three-Nation Agreed Declaration translated the notion of safeguards into the nuclear discourse. Yet, for the time being, it remained open how these safeguards would operate, which technical tools they would use, on which legal foundations they would be based, and which institution would administer them.^17^ The core idea was to tame the atom’s destructive aspects and foster its peaceful applications, granting non-nuclear weapon states access to the civilian uses of nuclear technology whilst inhibiting the spread of nuclear weapons to these states. These opposing goals of sharing and denial have weighed against each other in different ways over the last 70 years, and international nuclear safeguards became a key tool in handling the atom’s dual character. In 1946, UNAEC was the first forum in which diplomats from East and West discussed the issue of nuclear safeguards. The commission was composed of the permanent and non-permanent members of the Security Council; in addition, Canada was a member of UNAEC. However, as in the Security Council, Cold War tensions hindered any real progress in this forum.^18^

In the context of a new global order, nuclear safeguards appeared as a tool of verification not as a means of control. Using a set of different technical tools, accounting procedures, and on-the-ground inspections, they were envisaged as an external monitoring mechanism through which the international community would be able to gather insights into national nuclear programmes. The Western member-states of UNAEC supported the idea of nuclear safeguards. However, there was the prevailing understanding that safeguards alone were not enough to prevent the emergence of new nuclear weapon states. The American Baruch Plan, presented to the UNAEC in June 1946, was an expression of this scepticism toward the non-proliferation power of safeguards. Named after the man who presented it to the UNAEC, the American delegate and presidential advisor Bernard Baruch, its basis largely came from the work of the American expert committee that produced the March 1946 Acheson-Lilienthal Report.^19^ This latter effort, written in large part by Robert Oppenheimer, recommended not leaving “dangerous” nuclear activities in national hands. Baruch explained that, “no system of safeguards that can be devised will of itself provide an effective guarantee against production of atomic weapons by a nation bent on aggression.”^20^ Whilst international safeguards were part of Baruch’s plan to manage the atom’s dual character, they were just a supplement to control mechanisms. Rather than relying on safeguards, Baruch proposed the creation of an International Atomic Development Authority [IADA] that would have “managerial control or ownership of all atomic-energy activities potentially dangerous to world security,” including the production and ownership of fissionable materials, and research on nuclear explosives.^21^ Whilst fostering the peaceful uses of nuclear energy, the IADA would have the power to control all nuclear activities and impose sanctions on violators. It would not be subject to the veto of any state. Baruch continued: “If I read the signs aright, the peoples want a program not composed merely of pious thoughts but of enforceable sanctions—an international law with teeth in it.”^22^ Although the Baruch Plan supported peaceful nuclear technologies, it prioritised denial over sharing as it placed all potentially dangerous nuclear activities under international management or ownership.^23^

The Soviet counter-proposals to the American Baruch Plan remain largely neglected by existing literature.^24^ Interestingly, they anticipated the later shift from “denial over sharing” to “sharing over denial.” The Soviet ambassador to the UN, Andrei Gromyko, opposed the Baruch Plan for a number of reasons. He argued that an international authority as proposed by the Americans must be subject to the veto of the UN Security Council—a thought that Baruch had declined. The Soviets also feared that the United States would dominate the IADA. Notably, Gromyko criticised the Baruch Plan because it put the establishment of an international control system before the prohibition of nuclear weapons. The Soviets, whilst working secretly toward their own nuclear capabilities, argued publicly that the abolition of existing American atomic bombs had to precede the establishment of international control. Gromyko therefore introduced a different proposal, suggesting that nuclear activities of states should remain the responsibility of individual nations, rather than falling under the authority of an international body such as the IADA.

However, according to the Soviet plan, an international commission would carry out periodic inspections of national nuclear facilities and, if suspecting a state of violation, had authorisation to conduct additional special inspections as well. Because the Soviet UNAEC proposals of 1947 relied so heavily on on-the-ground inspections by an international commission, the leading French physicist and IAEA veteran, Bertrand Goldschmidt, later interpreted them as “a forerunner to the NPT.”^25^ At the time, however, the Western nations in the UNAEC perceived the safeguards provisions suggested by the Soviets as inadequate, pointing out that they would not be able to detect clandestine nuclear weapon programmes. Thus, in the mid-1940s, the Western states represented in the UNAEC viewed the very provisions that would become the key verification element in the later non-proliferation regime as insufficient. Furthermore, 20 years before the NPT negotiations, they thought it was “completely unrealistic to expect any nation to renounce atomic weapons without any assurance that all nations will be prevented from using them.”^26^ East–West disagreement over the future of the global nuclear order curbed any tangible progress, and after the Soviet Union developed and tested its own nuclear device in 1949, UNAEC became deadlocked and formally dissolved in 1951.

The American president’s, Dwight D. Eisenhower’s, “Atoms for Peace” speech, delivered to the UN General Assembly on 8 December 1953, was the next major initiative to strengthen the foundations of global nuclear order.^27^ At the time of the speech, the background for international diplomacy on nuclear matters had changed. The Soviet dictator, Joseph Stalin, was dead, a fact that promised to ease some of the tensions in Soviet–American diplomacy. On the other hand, the Soviet Union had developed and tested its own hydrogen bomb, a fact that underscored that the nuclear arms race was underway and accelerating. In light of the failed UNAEC negotiations a few years before, Eisenhower dismissed the earlier American plans to establish an IADA. Instead, he proposed the creation of a new international organisation, the IAEA, which would control an international pool of demilitarised fissionable materials. The basic task of the new Agency seemed simple: the nuclear weapon states and those with advanced nuclear programmes would transfer fissionable and other nuclear materials to the IAEA that, in turn, would make these materials available to its member-states for civilian nuclear applications in medicine, agriculture, science, and power generation. Like the Baruch Plan, “Atoms for Peace” had both sharing and denial aspects. In addition, the pool concept was an arms control proposal as it promised to reduce the stockpiles of the nuclear weapon states—at that time, the United States, Britain, and the Soviet Union. Strikingly, whilst with “Atoms for Peace” Eisenhower proposed the creation of the IAEA—an organisation that today is famous for its inspectorate—the original speech did not mention external safeguards on national nuclear programmes as one of the new Agency’s activities.^28^ Quite the contrary, before the UN General Assembly, Eisenhower emphasised that his plan to establish an international nuclear pool had “the great virtue that it can be undertaken without the irritations and mutual suspicions incident to any attempt to set up a completely acceptable system of worldwide inspection and control.”^29^

Why did Eisenhower’s “Atoms for Peace” speech result in the creation of an international organisation whose selling point was nuclear safeguards, whilst the original speech had other plans? The answer lays in both domestic and international contexts. “Atoms for Peace” was in large part inspired by Eisenhower’s own ideas and, before presenting it to the UN, he had only discussed it with his innermost group of advisors. The United States Atomic Energy Commission [AEC] was, apart from its chairman, Lewis Strauss, left out entirely. After the president’s appearance before the UN, the Americans exchanged views on “Atoms for Peace” with the Soviets, who initially had strong reservations toward the proposal.^30^ As long as the Soviets had not pledged to participate in the new Agency, any plans to establish a pool of fissionable materials, contributed from the stockpiles of the states principally involved, lost momentum; without Soviet participation, siphoning off fissionable materials from military stockpiles did not make much sense to the United States. Already during the planning stage of “Atoms for Peace,” Strauss had emphasised the lack of precise knowledge about the Soviet nuclear programme and argued that the development of the more destructive hydrogen bomb had reduced “the relative importance of a stockpile of fissionable material.”^31^ The dialogue with the Soviets made the involved responsible desks in the State Department also realise other difficulties with the presidential initiative. They concluded that the “Atoms for Peace” proposal had been developed and released without considering the risks involved in the spread of nuclear materials and technology.^32^

In light of these considerations, the Americans suggested that the two governments hold joint meetings to discuss how nuclear safeguards could ensure that states were not diverting the nuclear materials provided through the new Agency from civilian to military uses. In late August 1955, in the days following a huge UN Conference on the Peaceful Uses of Atomic Energy, the Americans and Soviets undertook a series of meetings to discuss these safeguards against diversion of nuclear materials. The meetings took place in UN European headquarters at Geneva, but—in opposition to the UNAEC and Eisenhower’s speech—they were not an official UN event. The organisation was neither involved in the meetings nor was its Secretariat informed about their progress. These meetings centred on how to ensure that “nuclear material for use in reactors can be supplied to members of an International Atomic Energy Agency without increasing the risk to the security of the world.”^33^ Delegates from Canada, France, Britain, and Czechoslovakia also participated in these so-called “meetings of six governments,” and the participants from both sides of the Iron Curtain mostly knew each other for years, having met regularly in different international fora. Both the Eastern and Western press described the closed talks as “ultra-secret”; the participation of eminent scientists such as the American, Isidor I. Rabi, and the Soviet, Dimitri Skobeltzyn, added to the public perception that the talks were highly important.^34^ Rabi, as the chief American delegate, announced in the first session that the meetings were designed “to talk about a technical problem in a technical manner,” a promise largely kept by the participants. Rabi argued that whilst the drafting of the IAEA statute was “a matter for diplomats and lawyers,” the scientists would have to deal with the technical feasibility of inspection and control.^35^

The larger political questions such as disarmament or the conditions for Soviet participation in the IAEA were not on the agenda. Although the atmosphere of the Geneva meetings was business-like, American and Soviet delegates were unable to reach an agreement on a technical solution to the “diversion problem.” The American delegation had come up with a half-baked proposal, drafted on short notice in a Geneva hotel room, to use radioactive tracers, such as high-energy gamma emitters, to track the movement of any nuclear fuel. The Americans blamed the Soviets for coming unprepared to the meeting, but they were also having difficulty figuring out which verification mechanisms made sense. Although ending without any concrete technical fix for the safeguards against diversion, the conversations with the Soviet scientists sharpened American understanding of the potential hazards of nuclear weapons proliferation and some of the available tools to address them. For the first time, experts from different countries discussed together safeguards objectives and procedures such as inspection and auditing. A State Department official, Gerald Smith, would later say that he felt that at that time, “if only American scientists and our Soviet counterparts could have exchanges about controlling nuclear weapons, the chances would be better than if it were left to politicians, bureaucrats, and military officers.”^36^

The distinction between what is technical and what is political became very important for the IAEA and would be a feature of many disputes during the Agency’s next 60 years. The IAEA has always prided itself in being a technical, not a political institution and framed safeguards as a technical solution to political problems.^37^ However, throughout the IAEA’s history, this separation between the political and technical realms has been largely artificial. It was already salient in the Geneva meetings that were ostensibly purely technical in nature. As the discussions progressed, it became clear that safeguards had strong political implications. This led to the understanding that only the IAEA could apply safeguards to materials that it provided; that was a way to de-politicise the control system. As Rabi explained:

In other words, there is no political tinge in this in the sense that if the country asks for a kilogram of enriched material it thereby binds itself to be thoroughly supervised. We are simply speaking of a contract which has been made, which was specified, and the technical side of the inspection system is to apply to what has been contracted for.^38^

The talks with the Soviets revealed that the two Cold War enemies had more in common than either had expected. In the weeks after the meetings, the State Department’s Atomic Energy Section paid unprecedented attention to the potential security dangers of the “Atoms for Peace” plan. The group realised that “diversion” was not the only danger involved in the spread of nuclear weapons to other states. Baruch was especially concerned about how much the actual control of peaceful nuclear uses had “dropped out of consideration” in American arms control circles.^39^ Rabi felt that before the United States implemented peaceful nuclear assistance programmes, it was important to develop an appropriate system of verification and control, either directly by the United States or through the future IAEA. In a memorandum of September 1955, Smith summarised Rabi’s position: “Rabi said that we must get these controls working before our reactors are constructed abroad. He believed that even a country like India, when it had some plutonium production, would go into the weapons business.”^40^ By the mid-1950s, the State Department was clearly worried about the proliferation risks of plutonium production in dual-use reactors and gaseous diffusion technology for uranium enrichment, and it realised that “Atoms for Peace” could increase the problem.^41^ The working group in the State Department also recognised that the diffusion of knowledge and technology could become as dangerous as the diversion of fissile materials.

A report finished by Smith at around the same time clearly stated, “the Atoms for Peace program will result in a number of countries having competence in atomic energy technology at an earlier date than if such program did not exist.”^42^ Even more bluntly, and two years before the IAEA was established, this State Department report came to the conclusion that “Atoms for Peace” increased the risk of nuclear weapons proliferation. It stated, “the important question of control and disposition of plutonium formed in power reactors abroad has not been considered by the US.”^43^ The State Department thus was aware of the proliferation risk of breeder reactors, which produce more fissile material than they consume. A wide range of other aspects was discussed, amongst them the usefulness of sanctions, the role of “auditors” in the inspection process, the vulnerability of different reactor types to diversion, and the potential scope and mandate of safeguards.

Whilst scientists dominated the Geneva safeguards talks, the next level of the IAEA negotiations had a more political character, with mostly senior career diplomats participating. From February to April 1956, a group of 12 nations, including the Soviet Union, met in Washington, DC to draft the statute of the IAEA.^44^ The sessions occurred in private in offices of the State Department, with no press attending and no records released. This helped to keep Cold War propaganda largely out of the meetings. With hosting the meetings in the nation’s capital, the United States underlined its claim for leadership in the new organisation.

Yet, the meetings showed that America was willing to find common ground with its Cold War enemy. American and Soviet delegates praised the spirit of good co-operation and the cordial atmosphere of the meetings.^45^ In the final session, they voted on the IAEA statute as a whole rather than on individual articles, some of which still caused controversy. This shrewd move, suggested by the Soviet delegate, Gregory Zarubin, meant that the group demonstrated unanimity. The negotiators knew that they had to find consensus to have the support of the international community, which would have to approve the final text at a UN conference later that year. It was the beginning of the thermonuclear age, and the superpowers shared a true interest in finding agreement on nuclear matters. Because the developing nations had responded enthusiastically to Eisenhower’s promise of global access to nuclear energy, the Cold War enemies became partners and competitors alike.^46^

Nevertheless, the Soviet attitude toward nuclear safeguards remained ambivalent. They were not entirely dismissive of the concept of nuclear safeguards, and they supported the draft statute despite its safeguards provisions. However, the Soviets also supported young nations’ criticism that safeguards presented a new form of superpower colonialism. As a result, the Soviet and Indian delegations were able to weaken somewhat the immediate power of safeguards by decoupling IAEA membership and acceptance of safeguards.^47^ Next to the safeguards article, the final IAEA statute still allowed for the implementation of the original proposal of a neutralised pool of fissile materials that, however, only received new momentum with the plans for the establishment of a multilateral fuel bank of low-enriched uranium in Kazakhstan, inaugurated in August 2017.^48^ Importantly, the IAEA statute did not address the clandestine development of nuclear weapons, a risk that the UNAEC had clearly identified.^49^ Accordingly, the Agency’s first Inspector General, Allan McKnight, described the development from the Baruch Plan to the creation of the IAEA as “a shift in emphasis from control, with the dissemination of nuclear science and technology as an adjunct, to a proposal for promotion of peaceful uses, with control as an adjunct.”^50^ Whilst recent political science literature has paid a lot of attention to the question of whether or not the United States has been consistent in its non-proliferation policy, this literature has largely neglected the decisive shift identified by McKnight and other contemporaries.^51^

Contrary to today’s prevailing perception of the IAEA—at least in the United States and Europe—technical assistance and not safeguards were the Agency’s original selling point. The developing nations had especially high expectations in receiving aid in the use of nuclear radioisotopes and technologies. The Soviet Union largely supported these countries’ criticism of safeguards, and the Western European countries favoured “self-inspection” amongst the European Atomic Energy Community [Euratom] member-states over Agency safeguards.^52^ Even within the IAEA, nuclear safeguards were widely seen as a “disruptive element,” especially by the offices concerned with technical assistance.^53^ The first IAEA model agreement for safeguards, released in 1961, INFCIRC/26, therefore applied only to technical assistance relating to research and small power reactors—less than 100 megawatts—and to materials placed voluntarily under IAEA safeguards.^54^ Agency inspectors carried out the first inspection only in 1962 at a Norwegian research reactor.^55^

When, in 1970, the NPT made safeguards mandatory for all non-nuclear weapon states that had signed the treaty, it was not a sudden change but the result of a transformation period that began after the 1962 Cuban Missile Crisis and gained momentum after the first Chinese nuclear test in 1964. The NPT aimed to freeze the number of nuclear weapon states and limit the nuclear weapons club to those five states that had manufactured and exploded a nuclear device by January 1967. The nuclear weapon states pledged not to share nuclear weapons or related technology with the non-nuclear weapon states, whilst the latter promised not to seek these weapons. Whilst there is a growing body of literature on the diplomatic negotiations in Geneva that drafted the treaty—the Eighteen-Nation Disarmament Committee [ENDC] at the UN—scholars have largely neglected the negotiations that followed at the IAEA once the treaty had entered into force.^56^

Decisive steps toward the conclusion of the NPT happened in Vienna, at the IAEA’s Board of Governors and its Scientific Advisory Committee, and not in Geneva. Roland Timerbaev, a Soviet career diplomat with long and distinguished experience in arms control and non-proliferation, says that around 1963 and 1964, “Russia became more Catholic than the Pope of Rome, in terms of safeguards.”^57^ Fears that West Germany might develop a nuclear weapon programme and worries over China intensified Soviet fears and led to increased support for IAEA safeguards. Timerbaev recalls this as a time of “very active, very constructive” superpower co-operation regarding safeguards in the IAEA’s most powerful diplomatic forum. This working co-operation in the IAEA also facilitated productive dialogue in Geneva, where the Americans and Soviets reached many agreements during informal bilateral meetings, sometimes on a hike in the mountains near Geneva or a trip on a yacht.^58^ Especially, the Western industrialised nations and members of the non-aligned movement perceived these informal discussions as what Andrew Coe and Jane Vaynman have called “superpower collusion” over the NPT.^59^ “I saw no Cold War,” summarises the Egyptian diplomat, Mohamed Shaker, in his recollections of the ENDC negotiations in Geneva.^60^ In 1965, the IAEA model safeguards agreement, INFCIRC/66, replaced the earlier agreement of 1961 and, for the first time, enabled IAEA safeguards for inspecting large reactors. The model agreement defined the circumstances requiring safeguards as well as safeguards procedures. It was a breakthrough for the safeguards system. In the middle of the Cold War, both the IAEA Board of Governors and its General Conference endorsed INFCIRC/66 unanimously. Today, the IAEA’s inspections in Israel and Pakistan—two countries that have not signed the NPT—still find basis on this 1965 model agreement.

On 5 March 1970, the day the NPT entered into force, the Agency’s new Inspector General, the Swiss Rudolph Rometsch, reminded journalists at an IAEA press conference that the conclusion of the treaty did not mean “that the Agency will swing into action next Monday, spreading a net of safeguards over the whole world.”^61^ The drafters of the NPT had charged the IAEA with a new and far-reaching responsibility since each non-nuclear weapon state that had signed the treaty would be subject to nuclear safeguards. The Agency would have to apply safeguards “on all source or special fissionable material in all peaceful nuclear activities” within the territory of the respective member-state.^62^ These new “comprehensive safeguards” presented a major extension of the IAEA’s traditional safeguards system, which was mandatory only for recipients of technical assistance from the Agency and only applied to specified facilities. The NPT’s safeguards article had left the specifics of its implementation vague. In addition, and in line with the IAEA’s traditional safeguards, the NPT’s safeguards obligations were not self-enforcing.

To answer the remaining questions, the IAEA’s Swedish Director General Sigvard Eklund established a new diplomatic committee—the Safeguards Committee or Committee 22—in 1970, the deliberations of which in many ways presented a renegotiation of the NPT’s aims and objectives. The Safeguards Committee convened from June 1970 to March 1971. Its negotiations offer important insights into how, at a time of both rising hopes in nuclear power generation and growing fears of the spread of nuclear weapons, nuclear experts envisioned a global non-proliferation regime. The committee laid out principles that would shape safeguards activities and cultures for decades to come. Interestingly, the committee was open to all IAEA member-states, not just to members of the Board of Governors or signatories of the NPT. Even outspoken critics of the treaty with no or little intention to sign, such as representatives of Argentina, France, India, Israel, Pakistan, and South Africa, participated actively in the meetings and left their imprints on the new safeguards regime. Amongst the most committed participants were some of the leading experts on non-proliferation of the time, such as the long-standing American AEC official, Myron Kratzer, and Claude Zangger from Switzerland, who was about to become the Chairman of the Zangger Committee, a group of nuclear export nations. From the IAEA Secretariat, Eklund and Rometsch attended the meetings. However, Eklund interpreted his role as that of an officer, a servant to the member-states’ wishes. Safeguards related to “highly political” questions, and Eklund wanted to hew “to the principle that the Secretariat should act only on instructions from the governing bodies.”^63^

The line-up of states that left the most distinct imprints on the new safeguards regime included the United States and the Soviet Union, as well as the advanced industrial states of the West, notably the two big nuclear energy players, West Germany and Japan. The Soviet delegation based its input largely on theory; even though the Soviets had supported safeguards since around 1963, they lacked practical experience with the matter. At that time, Japan was the most experienced country in terms of having nuclear safeguards on its facilities, both in the framework of bilateral agreements and Agency agreements. The committee allowed West Germany to play a leading role because the German question was at the heart of the entire non-proliferation issue; it was critical that West Germany, widely regarded as a likely future proliferator, would ratify the treaty and conclude a safeguards agreement with the Agency.

Many meetings of the IAEA Safeguards Committee revolved around two fundamental questions that touched upon the very core of the NPT’s safeguards provisions. First, how would the IAEA know about the location and amount of “all source or special fissionable material in all peaceful nuclear activities” in signatory states? To put all material under safeguards was one thing, to know where all the material of a state was, another. Second, what did the negotiators expect comprehensive safeguards to accomplish? Were they designed to prevent the emergence of new nuclear weapon states or solely to detect eventual violations once they had taken place? As for the first question—about how the Agency would know “where to look”—there was a wide range of options on how to learn about member-states’ nuclear activities, ranging from the use of intelligence information to relying entirely on member-states’ own declarations. Kratzer was the most outspoken advocate for providing the IAEA with as much information as possible—a position with which he did not succeed. Despite these individual calls for broad access to information, the final guidelines—published as INFCIRC/153—underlined not only the importance of protecting information, but also emphasised that “the Agency shall require only the minimum amount of information and data consistent with carrying out its responsibilities under the Agreement.”^64^

The way the participating diplomats solved some of the fundamental differences in views had a lasting impact on the IAEA’s safeguards regime, as well as on “safeguards culture.”^65^ Because of the controversy over the use of information, the decision emerged that the IAEA would rely on its member-states’ declarations about their nuclear activities. However, when looking at the negotiations, it showed that Committee 22 agreed that the IAEA would have the right to scrutinise state declarations. This became evident already in the first set of meetings, when the South African delegation introduced a proposal that aimed to deny the Agency’s safeguards any legal means to access information beyond the declarations of states. The South Africans proposed, “that the safeguarding and inspection functions of the Agency … shall be concerned solely with the material reported upon by the State concerned to the Agency in the initial and subsequent reports and materials derived therefrom.”^66^ In plain English, this proposal suggested that it was not the IAEA’s business to look beyond state declarations or doubt their correctness. The Hungarian delegate, Otto Lendvai, found sharp words to express his opposition to the proposal, which he believed would steal much of the NPT’s thunder. Relying entirely on state declarations “would be tantamount to allowing the State to decide unilaterally what materials it wished to declare to the Agency.”^67^ The majority of other delegations in the Safeguards Committee also rejected the South African proposal. This course was somewhat characteristic for the negotiation history of the guidelines for the new “comprehensive safeguards.” Controversial issues remained open for future interpretation—interestingly and importantly, the negotiators decided to delete the definition of the key word “verification” from the INFCIRC/153 glossary. Despite these omissions, INFCIRC/153 spelled out some far-reaching rights for Agency safeguards to obtain information; notably, they would have the right to conduct special inspections in member-states when routine inspections pointed to irregularities. The participants of the Safeguards Committee realised that special inspections, in the words of the German delegate, Werner Ungerer, “surely represented the most delicate area in the whole inspection process.”^68^ Whilst the instrument of special inspections carried some ammunition as it put additional pressure on states, the later practice showed that this instrument was almost never used.^69^

What were comprehensive safeguards under the NPT actually expected to achieve? Sir Alex Baxter, the Australian representative, underlined that there was a crucial gap between what the NPT aimed to achieve and what the IAEA could do in supporting the treaty’s objectives. Baxter explained, “though the purpose of the Treaty was to prevent proliferation of nuclear weapons by any means, the application of the Agency’s safeguards … would have the sole objective of preventing nuclear energy in non-nuclear-weapon States from being diverted from peaceful uses to the production of nuclear weapons or other nuclear devices.”^70^ In other words, Agency safeguards could not prevent the proliferation of nuclear weapons but just prevent the illegal use of nuclear materials. Baxter’s counterpart from Italy went a step further and argued that the application of Agency safeguards could not even prevent the diversion of nuclear materials; it could only verify that member-states did not divert nuclear materials—and detect an eventual diversion—but not stop a state from diverting nuclear materials.^71^ So if the delegations agreed that safeguards could not prevent nuclear weapons proliferation, what was their objective? How would they serve the ends of the NPT, which by its very name, was a non-proliferation treaty?

The discussions in the IAEA Safeguards Committee reveal that the drafters of comprehensive safeguards did not believe that safeguards alone were able to prevent nuclear weapons proliferation but, rather, would be a confidence-building measure that primarily had a deterring effect. Safeguards were to “detect and thereby deter.”^72^ Whilst, according to Article III of the NPT, the IAEA would apply safeguards “with a view to preventing diversion of nuclear energy from peaceful uses to nuclear weapons or other nuclear explosive devices,”^73^ INFCIRC/153 gave a watered-down version of what safeguards could achieve. The objective of safeguards was “the timely detection of diversion of significant quantities of nuclear material … and deterrence of such diversion by the risk of early detection.”^74^ The concept of deterrence by detection offered a weak solution in line with the limited powers granted to the IAEA. What would deter states? First, the negotiators believed that the risk of exposure as a violator of the treaty would deter states from illegally diverting nuclear materials. Second, the safeguards article of the IAEA statute rooted deterrence in the denial of Agency resources, assistance, and participation. “In the event of non-compliance,” the Agency had the right “to suspend or terminate assistance and withdraw any materials and equipment made available by the Agency or a member.” In addition, the Agency could also “suspend any non-complying member from the exercise of the privileges and rights of membership.”^75^ Finally, the IAEA would also report cases of non-compliance to the Security Council of the UN.^76^ The IAEA had no power to punish signatories of the NPT, let alone means to coerce states to adhere to the NPT. Whilst, in March 1971, a Central Intelligence Agency [CIA] memorandum still called the IAEA the “NPT’s enforcement agency,” over the course of the Safeguards Committee’s deliberations it had become clear that it had no real enforcement power.^77^

Next to the deterrence framing, safeguards appeared as a means to build confidence between member-states. In the first round of meetings, when the heads of the participating states outlined their overall visions for future nuclear safeguards, many of them centred their remarks on the idea of confidence. The recurring use of the expression “climate of confidence” was very prominent. Primarily the industrialised nations of the West and the producers of source material framed nuclear safeguards as a confidence-building measure—precisely those states that were afraid that strengthened nuclear safeguards would interfere in national matters and present a potential threat to industrial secrets. The South African Donald Sole, who had been involved in IAEA matters since the organisation’s inception, saw this kind of “climate of confidence” between member-states as “absolutely essential.” The West German delegate also used the phrase, as did the Italian, Turkish, and Swiss delegates, to name just a few examples.^78^ The South African delegate made clear that his country would not support safeguards that would be operating like a “police force.”^79^ The Japanese delegate, in summarising his governments’ position in the meetings, said that Agency safeguards were “different from internationally-sponsored spy activities … and should not be conceived of as Big Brother’s watch over everybody’s shoulder.”^80^

The “climate of confidence” thus became a formula to support the idea of a safeguards system that relied mainly on member-states’ declarations and was not intrusive. The delegate from Spain, Jesus Riosalido, argued, “the idea of an inventory drawn up by the States themselves was evidence of great confidence in the latter; one could not but welcome that demonstration of confidence.”^81^ The climate of confidence would also shape relations between IAEA inspectors and reactor operators in member-states: “the frequency with which audit samples are analysed will depend on the mutual confidence established between operator and inspector.”^82^ Political analysts of the time already feared that this might hinder good inspection. As the political scientist George H. Quester warned as early as 1970, nuclear safeguards on national levels had shown that there was a “tendency of regulatory agencies to become cooperative (or even overly cooperative) with the industries regulated.”^83^ However, only in the 1980s did this lead to the introduction of a rotation policy for safeguards inspectors at the Agency.^84^ On the other hand, in the 1970s, there was also the belief that inspectors would not find many instances of diversion and, accordingly, safeguards inspectors might find their job boring: “Watching for violations will be dull work in any event, for there may well never be any to detect.”^85^

When the Safeguards Committee ended its work, the participants celebrated it as a major success. The German governor, Ungerer, even honoured the newly drafted safeguards guidelines with an impromptu music composition. Sitting at the piano, he played a song about the safeguards negotiations, culminating in the optimistic chorus “We agree!”^86^

The IAEA was not initially a non-proliferation agency. Furthermore, the establishment of the Agency’s comprehensive nuclear safeguards regime was the result of a two-decade long international negotiation process and not the original motivation to launch this international organisation. Over the past 60 years, nuclear safeguards have undergone several re-framings. Whilst in the beginning of the nuclear age, they were widely perceived as necessary but too weak to prevent nuclear weapons proliferation on their own, they later became the key verification tool of the non-proliferation regime. When the IAEA Safeguards Committee translated Article III of the NPT into concrete guidelines and procedures in 1970 and 1971, this was partially a renegotiation of the NPT and in fact limited its force. Whilst the participants realised that nuclear safeguards could not prevent the spread of nuclear weapons, they tried to frame this shortcoming as something positive: a confidence-building measure as opposed to an intrusive police force. The negotiations also confirmed what had crystallised already in the 1955 Geneva technical talks: that IAEA safeguards would limit themselves to the detection of diversion—and not look for clandestine weapon programmes. At the same time, and whilst the tradition of IAEA safeguards in fact focused on the detection of eventual diversion from declared nuclear programmes, the negotiators of the IAEA statute left the door open to more powerful safeguards, and even to “anytime, anywhere” access for IAEA inspectors should this become part of a safeguards agreement.

The statute’s safeguards article granted the IAEA

the right to send into the territory of the recipient State or States inspectors, designated by the Agency after consultation with the State or States concerned, who shall have access at all times to all places and data and to any person who by reason of his occupation deals with materials, equipment, or facilities which are required by this Statute to be safeguarded.^87^

The proliferation crises of the early 1990s—South Africa, North Korea, Iraq—have led to the strengthening of IAEA safeguards, visible in the conclusion of the Additional Protocol of 1997. Controversial questions, first discussed in the 1970 Safeguards Committee, were taken up again and member-states now decided to give the IAEA more access to information—for example, by using intelligence information and environmental sampling. When, in June 2016, the cover of the IAEA Bulletin featured the motto “IAEA Safeguards: Preventing the Spread of Nuclear Weapons,” this was the expression of a new understanding of the role of safeguards in the global nuclear order.^88^

## Notes

1. For the concept of “global nuclear order” and an overview of the related literature, see Nicola Horsburgh, *China and Global Nuclear Order: From Estrangement to Active Engagement* (Oxford: 2015), 17–38.

2. See, for example, the definitions of nuclear safeguards by the IAEA and the British Office of Nuclear Regulation (2017): .https://www.iaea.org/topics/safeguards-and-verification, http://www  onr.org.uk/safeguards/what.htm.

3. Mohamed El Baradei, *The Age of Deception: Nuclear Diplomacy in Treacherous Times* (London: 2011), 37–47.

4. On the JCPOA, see the IAEA website: https://www.iaea.org/newscenter/focus/iran.

5. On the early history of IAEA safeguards, see Astrid Forland, “Negotiating Supranational Rules: The Genesis of the International Atomic Energy Agency Safeguards System” (PhD dissertation, University of Bergen, 1997); David Fischer, “Nuclear Safeguards: The First Steps,” *IAEA Bulletin* 49/1 (2007): 7–11; most recently, Leonard Weiss, “Safeguards and the NPT: Where our Current Problem Began,” *Bulletin of the Atomic Scientists* (21 August 2017): http://dx.doi.org/10.1080/00963402.2017.1362906; Jacob Darwin Hamblin, “A Glaring Defect in the System. Nuclear Safeguards and the Invisibility of Technology,” in *Negotiating the Nuclear Nonproliferation Treaty: Origins of the Nuclear Order*, edited by Roland Popp, Liviu Horowitz, and Andreas Wenger (London: 2017), 203–20. For a legal history perspective, see Paul C. Szasz, *The Law and Practices of the International Atomic Energy Agency* (Vienna: 1970), 531–657.

6. “Atomic Energy: Agreed declaration signed at Washington, November 15, 1945”: https://www.loc.gov/law/help/us-treaties/bevans/m-ust000003-1304.pdf.

7. The IAEA is an independent inter-governmental organisation linked to the UN through a separate agreement. On the creation of the IAEA, see Elisabeth Roehrlich, “The Cold War, the Developing World, and the Creation of the International Atomic Energy Agency (IAEA), 1953–1957,” *Cold War History* 16/2 (2016): 195–212.

8. Treaty on the Non-Proliferation of Nuclear Weapons, published as IAEA INFCIRC/140 (22 April 1970): https://www.iaea.org/sites/default/files/publications/documents/infcircs/1970/infcirc140.pdf. Apart from the international level, regional initiatives such as Euratom and the Brazilian-Argentine Agency for Accounting and Control of Nuclear Materials have introduced nuclear safeguards regimes as well.

9. For the history of the IAEA, see David Fischer, *History of the IAEA: First Forty Years* (Vienna: 1997); Lawrence Scheinman, *The IAEA and World Nuclear Order* (Washington, DC: 1987). On safeguards and bureaucracy, see especially Anna Weichselbraun, “Constituting the International Nuclear Order: Bureaucratic Objectivity at the IAEA” (PhD dissertation, University of Chicago, 2016).

10. These include notably the records of the Board of Governors, the Safeguards Committee, and the Scientific Advisory Committee.

11. Bernhard G. Bechhoefer, “Negotiating the Statute of the International Atomic Energy Agency,” *International Organization* 13/1 (1959): 38.

12. Dieter Goethel, “International Organizations During the Cold War: How the Superpowers Exploited Staffing Policies to Gain Control over the IAEA Secretariat,” unpublished paper for the workshop “The History of the IAEA During the Global Cold War,” Berlin Center for Cold War Studies (2 September 2016), 4.

13. I thank the Agency’s former director of personnel, Dieter Goethel, for pointing my attention to the IAEA Staff Regulations. For the current version, see INFCIRC/612 (30 August 2002): https://www.iaea.org/publications/documents/infcircs/staff-regulations-agency.

14. For the policy-making organs of the IAEA, see Szasz, *Law and Practices*, 107–226.

15. Laura Rockwood, “The IAEA and International Safeguards,” in *Routledge Handbook of Nuclear Proliferation and Policy*, edited by Joseph F. Pilat and Nathan E. Busch (London: 2015), 143–44.

16. Charles Howard Ellis, *The Origin, Structure and Working of the League of Nations* (Clark, NJ: 2004), 88.

17. For a history of these early postwar plans, see the chapter “Seeking International Control of Nuclear Weapons” in *A Global History of the Nuclear Arms Race: Weapons, Strategy, and Politics*, edited by Richard Dean Burns and Joseph M. Siracusa (Santa Barbara, CA: 2013), 67–88.

18. On the history of the UNAEC, see David W. Kearn Jr., “The Baruch Plan and the Quest for Atomic Disarmament,” *Diplomacy and Statecraft* 21/1 (2010): 41–67; Larry G. Geber, “The Baruch Plan and the Origins of the Cold War,” *Diplomatic History* 6/4 (1982): 69–95. For the early history of the UN, Ilya V. Gaiduk, *Divided Together: The United States and the Soviet Union in the United Nations, 1945–1965* (Palo Alto, CA: 2013).

19. Kearn, “Baruch Plan,” 48–51.

20. Baruch presented the plan to the UNAEC on June 14, 1946. Also see, Szasz, *Law and Practices*, 11–12.

21. Baruch, UNAEC, 14 June 1946.

22. Ibid.

23. In American nuclear law, this emphasis on secrecy and denial found its expression in the restrictive *Atomic Energy Act* of 1946.

24. An exception is Bertrand Goldschmidt, “A Forerunner of the NPT? The Soviet Proposals of 1947: A Retrospective Look at Attempts to Control the Spread of Nuclear Weapons,” *IAEA Bulletin*, Spring 1986, 58–64.

25. Ibid.

26. Ibid., 64.

27. For historical scholarship on Eisenhower’s “Atoms for Peace” speech, its aims, and consequences, see Ira Chernus, *Eisenhower’s Atoms for Peace* (College Station, TX: 2002) as well as John Krige, “Atoms for Peace, Scientific Internationalism, and Scientific Intelligence,” in *Global Knowledge Power: Science and Technology in International Affairs*, edited by John Krige and Kai-Henrik Barth, vol. 21 (Osiris: 2006), 16–81; Richard G. Hewlett and Jack M. Holl, *Atoms for Peace and War, 1953–1961: Eisenhower and the Atomic Energy Commission* (Berkeley, CA: 1989); Kenneth Osgood, *Total Cold War: Eisenhower’s Secret Propaganda Battle at Home and Abroad* (Lawrence, KS: 2006); Mara Drogan, “Atoms for Peace, US Foreign Policy, and the Globalization of Nuclear Technology, 1953–1960” (PhD dissertation, State University of New York, Albany, 2011). Also see the two edited volumes published on the occasions of the thirtieth and fiftieth anniversaries of “Atoms for Peace,” respectively Joseph F. Pilat, Robert E. Pendley, and Charles K. Ebinger, eds., *Atoms for Peace: An Analysis after 30 Years* (Boulder, CO: 1985); Joseph F. Pilat, *Atoms for Peace: A Future after 50 Years* (Washington, DC: 2005).

28. For the full text of Eisenhower’s speech, see Chernus, *Atoms for Peace*, xi–xix. On “Atoms for Peace” and arms control, see Henry Sokolski, “The Arms Control Connection,” in *Atoms for Peace*, edited by Pilat, Pendley, and Ebinger, eds., 35–50.

29. Chernus, *Atoms for Peace*, xvii.

30. David Holloway, “The Soviet Union and the Creation of the IAEA,” *Cold War History* 16/2 (2016): 177. The Soviet–American exchange on “Atoms for Peace” was published shortly afterwards: “Correspondence with Soviet Union on Atomic Pool Proposal,” *Department of State Bulletin* 31/797 (4 October 1954): 478–97; and “Correspondence with USSR on Peaceful Uses of Atomic Energy,” *Department of State Bulletin* 35/904 (1956): 620–31.

31. Strauss memorandum for the President, 17 September 1957, DDE [Dwight Eisenhower Papers President, Administration Series, Dwight D. Eisenhower Library and Museum, Abilene, KS] Box 5, Atoms for Peace, NAID #12022697.

32. Mara Drogan, “The Nuclear Imperative: Atoms for Peace and US Policy on Exporting Nuclear Power in the 1950s,” *Diplomatic History* 40/5 (2016): 948–74.

33. The scientist, Isidor Rabi, at the first meeting on 23 August 1955; for the verbatim records of the meetings see RG 326 Box 5. [International Affairs, IAEA: PV documents 1955, Records of the Atomic Energy Commission, Records Relating to the Formation of the International Atomic Energy Agency, 1953–1957, National Archives and Records Administration, College Park, MD] A brief overview of the history of the meetings is in Hewlett and Holl, *Atoms for Peace and War*, 313–15.

34. American Embassy [Paris] to USIA, 23 August 1955, RG 59, General Records Relating to Atomic Energy Matters, 1948–1962, Box 137, Proposed IAEA 1955.

35. Rabi at the first meeting on 22 August 1955.

36. Gerard C. Smith, *Disarming Diplomat: The Memoirs of Gerard C. Smith, Arms Control Negotiator* (Lanham, MD: 1996), 45.

37. Anna Weichselbraun, “Nuclear Experts at the IAEA: Safeguards Inspectors and Pre-NPT Verification Efforts,” conference paper at “The Global History of the IAEA,” organised by CISAC/Stanford University and the University of Vienna (18–20 September 2014).

38. Rabi at the second meeting, 23 August 1955.

39. “Control of Peacetime Uses of Atomic Energy,” 7 October 1955, RG 59 General Records Relating to Atomic Energy Matters, 1948–1962, File: IAEA Control and Inspection, 1955–1957, Box 137.

40. Smith “Memorandum for the File, subject: Discussion with Dr. Rabi—technical planning for IAEA,” 14 September 1955, RG 59 General Records Relating to Atomic Energy Matters, 1948–1962, File: IAEA Control and Inspection, 1955–1957, Box 137.

41. “Memorandum for the File,” 12 December 1955, Ibid.

42. “Observations on the problem of controlling against diversion of fissionable materials from nuclear reactors,” 17 September 1955, Ibid.

43. Ibid.

44. The IAEA Archives in Vienna hold copies of the official records of the Washington talks; see “Working Level Meeting on the Draft Statute of the International Atomic Energy Agency,” CS/OR 1–40 (20 September–26 October 1956), IAEA Archives.

45. James J. Wadsworth, “Modern Diplomacy: Atoms for Peace,” in *Power and Order: 6 Cases in World Politics*, edited by John G. Stoessinger and Alan F. Westin, eds. (NY: 1964), 34–35.

46. Roehrlich, “Creation of the IAEA,” 211. On the North-South dimension of the IAEA negotiations, see also Gabrielle Hecht, “Negotiating Global Nuclearities: Apartheid, Decolonization, and the Cold War in the Making of the IAEA,” in Krige and Barth, *Global Power Knowledge*, 25–48.

47. Lawrence Scheinman, *The Nonproliferation Role of the International Atomic Energy Agency: A Critical Assessment* (Washington, DC: 1985), 27. Also see Rockwood, “IAEA and International Safeguards.”

48. Tariq Rauf, “From ‘Atoms for Peace’ to an IAEA Nuclear Fuel Bank,” *Arms Control Today*, October 2015, https://www.armscontrol.org/ACT/2015_10/Features/From-Atoms-for-Peace-to-an-IAEA-Nuclear-Fuel-Bank; on the inauguration, see https://www.iaea.org/newscenter/pressreleases/iaea-leu-bank-reaches-milestone-with-storage-facility-inauguration-in-kazakhstan.

49. Allan McKnight, *Atomic Safeguards: A Study in International Verification* (NY: 1971), 14–15.

50. Ibid., 25.

51. On this debate, see Thomas Cavanna, “Geopolitics over Proliferation: the Origins of US Grand Strategy and their Implications for the Spread of Nuclear Weapons in South Asia,” *Journal of Strategic Studies* (10 June 2016): 1–28, http://www.tandfonline.com/doi/full/ 10.1080/01402390.2016.1184148; Francis J. Gavin, “Strategies of Inhibition: U.S. Grand Strategy, the Nuclear Revolution, and Grand Strategy,” *International Security* 40/1 (2015): 9–46; ISSF Article Review Forum 68 on Nonproliferation (6 February 2017): https://networks.h-net.org/node/28443/discussions/165195/issf-article-review-forum-68-nonproliferation-6-february−2017.

52. John Krige, “Euratom and the IAEA: The Problem of Self-Inspection,” *Cold War History* 15/3 (2015): 341–52.

53. Carlos Büchler, “Safeguards: The Beginnings,” in *International Atomic Energy Agency: Personal Reflections*, edited by David Fischer (Vienna: 1997), 49.

54. IAEA, “The Agency’s Safeguards,” INFCIRC/26, 30 March 1961.

55. On the IAEA’s first inspection, see PR [IAEA Press Releases, IAEA Archives, Vienna] 62/8.

56. On the most recent history of the NPT negotiations see the edited volume, Popp, Horowitz, and Wenger, *Nuclear Nonproliferation Treaty*.

57. Transcript of Roland Timerbaev interview [conducted by Rich Hooper and Jenni Rissanen], 14 June 2007 (PNNL-SA-56315), 5–6: http://cgs.pnnl.gov/fois/doclib/Timerbaev(transcript)2.pdf.

58. Ibid.; Transcript of George Bunn interview [conducted by Tom Shea and Danielle Peterson], 17 February 2006 (PNNL-SA-49240): http://cgs.pnnl.gov/FoIS/doclib/Bunn_Stanford.pdf.

59. Andrew J. Coe and Jane Vaynman, “Collusion and the Nuclear Nonproliferation Regime,” *Journal of Politics* 77/4 (2015): 983–97.

60. Mohamed Shaker at a workshop on the history of the IAEA, “The IAEA and the Global Cold War,” Berlin Center for Cold War Studies (2 September 2016).

61. “NPT: Now a Reality,” *IAEA Bulletin* 13/3 (1971): 3.

62. Article III of the NPT.

63. “Safeguards Committee: Official Record of the First Meeting” (12 June 1970), IAEA GOV.COM.22/OR.1, 5.

64. Article 8, “The Structure and Content of Agreements between the Agency and States Required in Connection with the Treaty on the Non-Proliferation of Nuclear Weapons” (June 1972): INFCIRC/153: http://www.vertic.org/media/assets/nim_docs/Treaty/nuclear/INFCIRC-153%20(en).pdf, https://www.iaea.org/sites/default/files/publications/documents/infcircs/1970/infcirc140.pdf.

65. Trevor Findlay, “The IAEA’s Nuclear Safeguards Culture: ‘Candy Concept’ or Powerful Prism?,” 2014, https://www.iaea.org/safeguards/symposium/2014/home/eproceedings/sg2014-papers/000194.pdf.

66. “Safeguards Committee: Official Documents” (22 June 1970), IAEA GOV/COM.22/8.

67. “Safeguards Committee: Official Record of the Seventh Meeting” (19 June 1970), IAEA GOV/Com.22/OR.6.

68. “Safeguards Committee: Official Record of the Fifty-Seventh Meeting” (3 December 1970), IAEA GOV/Com.22/OR/56.

69. In 1992, the IAEA Board Governors confirmed the IAEA’s right to conduct special inspections, but emphasised that “such inspections, as regards undeclared locations, are only expected to occur on rare occasions.” See: “Strengthening the effectiveness and improving the efficiency of the safeguards system,” GC(39)/17 (22 August 1995): https://www.iaea.org/About/Policy/GC/GC39/GC39Documents/English/gc39-17_en.pdf.

70. “Safeguards Committee: Official Record of the First Meeting” (12 June 1970), IAEA GOV.COM.22/OR.1, 14.

71. “Safeguards Committee: Official Record of the Third Meeting” (16 June 1970), IAEA GOV.COM.22/OR.3, 10.

72. Ryukichi Imai, “Safeguards: Five Views,” *IAEA Bulletin* 13/3 (1971): 6.

73. “Treaty on the Non-proliferation of Nuclear Weapons” (22 April 1970), IAEA INFCIRC/140: https://www.iaea.org/sites/default/files/publications/documents/infcircs/1970/infcirc140.pdf.

74. “The Structure and Content of Agreements between the Agency and States Required in Connection with the Treaty on the Non-Proliferation of Nuclear Weapons” (June 1972), INFCIRC/153.

75. IAEA Statute, Article XII, 7.

76. Benjamin N. Schiff, “Dominance without Hegemony: U.S. Relations with the International Atomic Energy Agency,” in *The United States and Multilateral Institutions: Patterns of Changing Instrumentality and Influence*, edited by Margaret P. Karns and Karen A. Mingst (Boston, MA: 1990), 41.

77. CIA Directorate of Intelligence, Intelligence memorandum, “The IAEA Enters a New Era,” 31 March 1971: CREST [CIAE Records Search Tool, National Archives and Record Administration, College Park, MD]: https://www.cia.gov/library/readingroom/docs/CIA-RDP85T00875R001100100033-9.pdf.

78. “Safeguards Committee: Official Record of the Second Meeting” (15 June 1970), IAEA GOV/COM.22/OR.2, 5; “Safeguards Committee: Official Record of the Third Meeting” (16 June 1970), IAEA GOV/COM.22/OR.3, 10, “Safeguards Committee: Official Record of the Fourth Meeting” (17 June 1970), IAEA GOV/COM.22/OR.4, 18.

79. “Safeguards Committee: Official Record of the First Meeting” (12 June 1970), IAEA GOV/COM.22/OR.1, 9.

80. Ryukichi Imai, “Safeguards: Five Views,” *IAEA Bulletin* 13/3 (1971): 6.

81. “Safeguards Committee: Official Record of the Sixth Meeting” (19 June 1970), IAEA GOV/COM.22/OR.6, 10.

82. SAF/410–2(1) to SAF/410–2(2), 1967–1968, Working Group I, IAEA Archives.

83. George H. Quester, “The Nuclear Nonproliferation Treaty and the International Atomic Energy Agency,” *International Organization* 24/2 (1970): 166.

84. Interview of the author with William Lichliter, Vienna (13 March 2015).

85. Quester, “Nuclear Nonproliferation Treaty,” 166.

86. David Fischer, *Stopping the Spread of Nuclear Weapons: The Past and the Prospects* (London: 1992), 88; Ungerer, “Five Views,” 10.

87. IAEA statute, Article XII, A.6.

88. “IAEA Safeguards: Preventing the Spread of Nuclear Weapons,” *IAEA Bulletin* 57/2 (2016), https://www.iaea.org/publications/magazines/bulletin/57-2.qq.

